# Epidemiological analysis of Group A streptococcus infection diseases among children in Beijing, China under COVID-19 pandemic

**DOI:** 10.1186/s12887-023-03885-7

**Published:** 2023-02-13

**Authors:** Hongxin Li, Lin Zhou, Yong Zhao, Lijuan Ma, Haihua Zhang, Yan Liu, Xiaoyan Liu, Jin Hu

**Affiliations:** 1 Department of Dermatology, Children’s Hospital Affiliated to Capital Institute of Pediatrics, Beijing, 100020 China; 2 Department of Clinical Laboratory, Children’s Hospital Affiliated to Capital Institute of Pediatrics, Beijing, 100020 China; 3 Department of Reproductive Medicine, Senior Department of Obstetrics & Gynecology, The Seventh Medical Center of PLA General Hospital, Beijing, China

**Keywords:** Group A streptococcus, *Emm* type, Superantigen, Antimicrobial resistance, Scarlet fever, Children, COVID-19 pandemic, China

## Abstract

**Background:**

Group A streptococcus is human-restricted gram-positive pathogen, responsible for various clinical presentations from mild epidermis infections to life threatened invasive diseases. Under COVID-19 pandemic,. the characteristics of the epidemic strains of GAS could be different.

**Purpose:**

To investigate epidemiological and molecular features of isolates from GAS infections among children in Beijing, China between January 2020 and December 2021. Antimicrobial susceptibility profiling was performed based on Cinical Laboratory Sandards Institute. Distribution of macrolide-resistance genes, *emm* types, and superantigens was examined by polymerase chain reaction.

**Results:**

114 GAS isolates were collected which were frequent resistance against erythromycin (94.74%), followed by clindamycin (92.98%), tetracycline (87.72%). *Emm12* (46.49%), *emm1* (25.44%) were dominant *emm* types. Distribution of *ermB*, *ermA*, and *mefA* gene was 93.85%, 2.63%, and 14.04%, respectively. Frequent superantigenes identified were *smeZ* (97.39%)*, speG* (95.65%), and *speC* (92.17%). *Emm1* strains possessed *smeZ*, *ssa*, and *speC*, while *emm12* possessed *smeZ*, *ssa*, *speG*, and *speC*. Erythromycin resistance was predominantly mediated by *ermB.* Scarlet fever strains harbored *smeZ* (98.81%), *spe*C (94.05%). Impetigo strains harbored *smeZ* (88.98%), *ssa* (88.89%), and *speC* (88.89%). Psoriasis strains harbored *smeZ* (100%).

**Conclusions:**

Under COVID-19 pandemic, our collections of GAS infection cutaneous diseases decreased dramatically. Epidemiological analysis of GAS infections among children during COVID-19 pandemic was not significantly different from our previous study. There was a correlation among *emm*, superantigen gene and disease manifestations. Long-term surveillance and investigation of *emm* types and superantigens of GAS prevalence are imperative.

## Introduction

*Streptococcus pyogenes* (GAS) is vital human pathogen responsible for a wide spectrum of infectious diseases, not only infection on skin and respiratory, but also invasive diseases, such as streptococcal toxic shock syndrome, necrotizing fasciitis as well as triggered autoimmune diseases [1, 2]. Human immunity to GAS may be related with disease manifestations after GAS infection [3]. Severe GAS infection diseases account for 18.1 million cases around the world, with 1.78 million new cases and 500,000 deaths every year [4, 5]. Li analyzed epidemiological characteristics and changes in incidence of GAS infection diseases in China after SARS outbreak. The yearly incidence was 2.44 cases per 100,000. Case-fatality ratios was 0.03 case per 1000 people. Significant seasonal features were May to June and November to December. Scarlet fever in children was high incidence and case-fatality [6]. Comparing with USA, GAS infection in China was usually presented in non-invasive GAS infection [7, 8]

Antibiotic resistance increases gradually, causing global concern [9]. Resistance of isolates to antibiotic varies in different countries and regions [10, 11]. In China, GAS was high frequent resistance to macrolides and clindamycin [12]. M protein is an important virulence factor of GAS coded by *emm* gene. Depending on variation of N-terminal, more than 250 *emm* types have been identified. Surveillance on GAS *emm* types in a long period can give a valuable clue for prediction of future *emm* clones [13]. The prevalent *emm* types vary over time in different countries and regions [14]. In China, in the year of 2011, *emm12* was the most prevalent type in scarlet fever, with high resistance to erythromycin, tracycline, and clindamycin. However, epidemiological characteristics of M protein changed with time [15].

Sixteen known sAgs have been identified in GAS, including *speA*, *speC*, *speG-M*, *smeZ*, *ssa*, *speQ*, and *speR* [16], responsible for GAS virulence and successful infection pathogenesis [17].

Researches on GAS epidemiological features have been attracted great attention around the world. Relationship among GAS infection diseases, *emm* types, and *sAgs* distribution has not been identified [18–20]. Because COVID-19 pandemic has changed our lifestyle, molecular characteristics of GAS isolated from Chinese children may be different.

In this study, we analyzed *emm* types, *sAgs*, and antimicrobial susceptibility resistance of GAS isolates as well as GAS infection categories to find differences among GAS infected cutaneous diseases before and under COVID-19 pandemic.

## Materials and methods

### Strain collection

Our patients were from outpatient department of Dermatology in Children’s Hospital, Capital Institute of Pediatrics in Beijing China. This study was approved by the Ethics Committee of the Capital Institute of Pediatrics. Between January 2020 and December 2021, 114 GAS isolates were recovered from throat swabs and skin infections. Throat and skin swabs were obtained from patients by two physicians for routine microbiologic analysis.

### Bacterial identification

The samples were incubated in a CO_2_ incubator at 37℃ for 24–36 h on Colombian blood plate (BD, USA). Morphologically suspected GAS colonies were confirmed by Gram’s staining and latex agglutination with the Streptococcus grouping kit (Oxoid, Basingstoke, UK).

### Antimicrobial susceptibility testing

The antibiotic susceptibility testing was performed for 10 antibiotics by K-B method. Protocols followed our previous study. Susceptibility of bacteria was determined by diameter of bacteriostatic ring and CLSI standard. *Streptococcus pneumoniae* ATCC 49,619 was used as control strain.

### DNA extraction

DNA extraction of GAS genome was performed according to the recommended method by the Center for Disease Control and Prevention.

### *Emm* genotypes

All isolates were performed *emm* genotypes according to protocols and recommendations of CDC. Sequence data were compared with *emm* typing database (https://www2.cdc.gov/vaccines/biotech/strepblast.asp).

### Erythromycin-resistance gene detection

Erythromycin resistance genes *ermB*, *ermA*, and *mefA* were performed for all isolates. Primer sequences for *ermB*, *ermA* and *mefA* were designed by Suvorov [10]. Protocol and reaction mixture followed our previous studies [21, 22].

### Superantigen detection

Eleven virulence genes, consisting of *speA*, *speC*, *speG*, *speH*, *speI*, *speJ*, *speK*, *speL*, *speM*, *ssa*, and *smeZ* were amplied by PCR with primers presented by Green [23]. Protocol and reaction mixture followed our previous study.

## Results

### Clinical data

One hundred fourteen isolates were received including throat samples (*n* = 84) and skin samples (*n* = 30). Of 114 isolates, 84 strains were collected from scarlet fever, 17 strains from impetigo, 10 strains from psoriasis, 1 strain from allergic purpura, and 2 strains from suppurative tonsillitis. 43 strains (37.72%) were recovered from girls, and 71 strains (62.28%) were from boys.. Patients aged from 22 days to 11 years old (median 6.25 years).

### Antimicrobial susceptibility testing results

All GAS isolates were sensitive to penicillin, ceftriaxone, cefotaxime, vancomycin, and cefepime. The highest rate of resistance was against erythromycin (94.74%), followed by clindamycin (92.98%), tetracycline (87.72%). Distribution of antimicrobial susceptibility was presented in Table 1.

**Table 1:** Antimicrobial susceptibility test of 114 isolates of GAS from 2020 to 2021, compared with our previous studies [21, 22]

Antibiotic	Susceptibility (n %)			Intermediate (n %)			Resistant (n %)		
	2020–2021	2019	2016–2017	2020–2021	2019	2016–2017	2020–2021	2019	2016–2017
Penicillin	114/100	271/100	297/100	0/0	0/0	0/0	0/0	0/0	0/0
Ceftriaxone	114/100	271/100	297/100	0/0	0/0	0/0	0/0	0/0	0/0
Levofloxacin	110/96.49	271/100	297/100	4/3.51	0/0	0/0	0/0	0/0	0/0
Cefotaxime	114/100	271/100	297/100	0/0	0/0	0/0	0/0	0/0	0/0
Vancomycin	114/100	271/100	297/100	0/0	0/0	0/0	0/0	0/0	0/0
Cefepime	114/100	271/100	297/100	0	0/0	0/0	0/0	0/0	0/0
Chloramphenicol	103/90.35	261/96.31	283/95.29	9/7.89	6/2.21	12/4.04	2/1.75	4/1.48	2/0.67
Tetracycline	6/5.26	23/8.49	16/5.39	8/7.01	11/4.06	13/4.38	100/87.72	237/87.45	268/90.23
Erythromycin	5/4.39	7/2.58	3/1.01	1/0.88	7/2.58	2/0.67	108/94.74	257/94.83	292/98.3
Clindamycin	5/4.39	30/11.1	8/2.69	3/2.63	3/1.11	2/0.67	106/92.98	238/87.82	287/96.6

### Erythromycin-resistant gene distributions

One hundred eight erythromycin resistance isolates were found. 107 erythromycin resistance isolates (93.86%) harbored *ermB* gene, 2 isolates (1.75%) harbored *ermA* gene, and 16 isolates (14.04%) harbored *mefA* gene. The distribution of erythromycin resistance genes in GAS isolates from different diseases is presented in Table 2.

**Table 2:** Distribution of erythromycin-resistant genes GAS isolates in different diseases, compared with our previous studies [21, 22]

Diseases	*ermB* (n/%)			*ermA* (n/%)			*mefA* (n/%)		
	2020–2021	2019	2016–2017	2020–2021	2019	2016–2017	2020–2021	2019	2016–2017
Scarlet fever (*n* = 84)	80/95.24	199/90.87	292/97.64	3/3.57	7/3.20	0/0	13/15.48	19/8.68	5/1.68
Impetigo (*n* = 17)	17/100	25/86.31	0/0	0/0	1/3.45	0/0	2/11.76	1/3.45	0/0
Psoriasis (*n* = 10)	8/80	16/84.21	0/0	0/0	2/10.53	0/0	1/10	2/10.53	0/0
Allergic purpura (*n* = 1)	1/100	1/100	0/0	0/0	0/0	0/0	0/0	0/0	0/0
Suppurative tonsillitis (*n* = 2)	2/100	0/0	0/0	0/0	0/0	0/0	0/0	0/0	0/0

### *Emm* molecular typing

The 114 isolates exhibited a high genetic diversity. 13 *emm* types accounted for 114 isolates. The majorities of cases were *emm12* (53), *emm1* (29), *emm12.19* (5), and *emm12.67* (5). Distribution of *emm* types and erythromycin-resistant genes in 114 GAS is presented in Table 3.

**Table 3:** Distribution of *emm* types and erythromycin-resistant genes in 114 GAS isolates, compared with our previous studies [21, 22]

*emm* types	*emm* subtypes	Count			*ermB* (n/%)			*ermA* (n/%)			*mefA* (n/%)		
		2020–2021	2019	2016–2017	2020–2021	2019	2016–2017	2020–2021	2019	2016–2017	2020–2021	2019	2016–2017
*emm1.0*		31	89	78	27/23.68	84/31	78/26.3	0/0	2/0.74	0/0	4/3.51	10/3.7	2/0.67
*emm12.0*	*emm12.0*	62	93	139	51/44.74	85/31.37	136/45.79	1/0.88	3/1.11	0/0	10/8.77	5/1.85	1/0.34
	*emm12.19*	5	22	27	5/4.39	21/7.75	27/9.09	0/0	1/0.37	0/0	0/0	1/0.37	0/0
	*emm12.21*	4	2	2	3/2.63	2/0.74	2/0.67	0/0	0/0	0/0	1/0.88	1/0.37	0/0
	*emm12.29*	3	0	0	3/2.63	0/0	0/0	0/0	0/0	0/0	0/0	0/0	0/0
	*emm12.37*	3	4	8	3/2.63	4/1.48	6/2.02	0/0	0/0	0/0	0/0	0/0	0/0
	*emm12.65*	2	0	0	1/0.88	0/0	0/0	1/0.88	0/0	0/0	0/0	0/0	0/0
	*emm12.67*	5	0	0	4/3.51	0/0	0/0	0/0	0/0	0/0	1/0.88	0/0	0/0
	*emm12.69*	2	13	2	2/1.75	12/4.43	2/0.67	0/0	0/0	0/0	0/0	0/0	0/0
*emm4.0*		2	4	1	1/0.88	2/0.74	1/0.34	1/0.88	1/0.37	0/0	0/0	1/0.37	0/0
*emm11.0*		1	0	0	1/0.88	0/0	0/0	0/0	0/0	0/0	0/0	0/0	0/0
*emm75.0*		3	8	8	3/2.63	7/2.58	8/2.69	0/0	1/0.37	0/0	0/0	0/0	0/0
*emm89.0*		3	2	4	3/2.63	1/0.37	4/1.35	0/0	0/0	0/0	0/0	0/0	0/0
Total		114	271^a^	297^a^	107/93.85	243/89.67	97/64	3/2.63	10/3.69	0/0	16/14.04	22/8.12	5/1.68

^a^Total number of cases in that year, some emm subtypes were not found in the year 2020-2021

Thirteen different *emm* types were identified. The most common *emm* types were *emm*12.0 (53/114, 46.5%), *emm*1.0 (29/114, 25.4%). The most prevalent *emm* subtypes in GAS strains from scarlet fever were *emm12.0* (37/114, 32.5%), *emm1.0* (25/114, 21.9%), *emm12.19* (5/114, 4.4%), and *emm12.67* (4/114, 3.5%). The most predominant *emm* subtype in impetigo was *emm12.0* (13/114, 11.4%). The most common *emm* subtypes from psoriasis were *emm12.0* (2/114, 1.8%), *emm12.29* (2/114, 1.8%), and *emm89.0 (*2/114, 1.8%). Distribution of GAS *emm* genotypes of strains in different diseases is presented in Table 4.

**Table 4:** Distribution (%) of GAS *emm* genotypes of strains in different diseases among children in Beijing from 2020 to 2021, compared with our previous studies [21, 22]

*emm* types	Scarlet fever			Impetigo			Psoriasis			Allergic purpura			Suppurative tonsillitis			Total		
	2020–2021	2019	2016–2017	2020–2021	2019	2016–2017	2020–2021	2019	2016–2017	2020–2021	2019	2016–2017	2020–2021	2019	2016–2017	2020–2021	2019	2016–2017
*emm1.0*	25	72	78	1	9	0	1	6	0	0	0	0	2	0	0	29	89	78
*emm12.0*	37	78	139	13	11	0	2	4	0	1	0	0	0	0	0	53	93	138
*emm11.0*	0	0	0	0	0	0	1	0	0	0	0	0	0	0	0	1	0	0
*emm12.19*	5	17	27	0	3	0	0	1	0	0	0	0	0	0	0	5	21	27
*emm12.21*	3	1	2	0	0	0	0	1	0	0	0	0	0	0	0	3	2	2
*emm12.29*	1	0	0	0	0	0	2	0	0	0	0	0	0	0	0	3	0	0
*emm12.37*	2	3	8	1	0	0	0	0	0	0	1	0	0	0	0	3	4	8
*emm12.65*	2	0	0	0	0	0	0	0	0	0	0	0	0	0	0	2	0	0
*emm12.67*	4	0	0	0	0	0	1	0	0	0	0	0	0	0	0	5	0	0
*emm12.69*	1	12	2	1	1	0	0	0	0	0	0	0	0	0	0	2	13	2
*emm4.0*	1	3	1	0	1	0	0	0	0	0	0	0	0	0	0	1	4	1
*emm75.0*	2	5	8	0	1	0	1	2	0	0	0	0	0	0	0	3	8	8
*emm89.0*	1	1	4	1	0	0	2	1	0	0	0	0	0		0	4	2	4
Total	84	219	297	17	29	0	10	19	0	1	1	0	2	0	0	114	271	297

### *Emm* types and superantigen distribution

Among 114 GAS isolates, the most predominant superantigen genes detected were *smeZ* (112/114, 98.25%), *speG* (110/114, 96.49%), and *speC* (106/114, 92.98%). Among 53 *emm12.0* isolates, the most prevalent superantigen genes detected were *speG* (51/114, 44.74%), *ssa* (51/114, 44.74%), and *smeZ* (51/114, 44.74%). Among 29 *emm1.0* GAS isolates, the most predominant superantigen genes detected were *speG* (29/114, 25.44%), *ssa* (29/114, 25.44%), *smeZ* (29/114, 25.44%), *speC* (29/114, 25.44%). and *speA* (24/114, 21.05%). The distributions of *emm* types and superantigens in GAS isolates is shown in Table 5.

**Table 5:** Distributions of *emm* types and superantigens in GAS isolates

*emm* Types	Distribution of superantigens (n)											
	Count	*specA*	*speC*	*speH*	*speI*	*speJ*	*speK*	*speL*	*speM*	*speG*	*ssa*	*smeZ*
*emm1.0*	29	24	29	5	3	27	0	0	1	29	29	29
*emm12.0*	53	4	49	45	43	3	0	0	2	51	51	51
*emm11.0*	1	0	1	1	1	0	0	0	0	1	0	1
*emm12.19*	5	1	5	0	0	2	0	0	0	5	5	5
*emm12.21*	3	1	3	2	2	1	0	0	1	3	3	3
*emm12.29*	3	0	3	3	3	0	0	0	1	3	3	3
*emm12.37*	3	0	3	3	3	0	0	0	0	3	3	3
*emm12.65*	2	0	2	0	0	0	0	0	0	1	2	2
*emm12.67*	5	1	3	4	4	0	0	0	0	5	3	5
*emm12.69*	2	1	2	0	0	1	0	0	0	2	2	2
*emm4.0*	1	0	1	0	0	0	0	0	0	0	1	1
*emm75.0*	3	0	3	3	3	0	0	0	3	3	0	3
*emm89.0*	4	0	2	0	0	0	1	0	0	4	1	4
Total	114	32	106	66	62	34	1	0	8	110	103	112
Percentage (%)		28.03	92.98	57.98	54.38	29.82	0.88	0	7.02	96.49	90.35	98.25

## Discussion

*Streptococcus pyogenes* is bacterial pathogen worldwide responsible for a broad spectrum of infection diseases as well as autoimmune sequelae [1, 4]. Epidemiological and molecular features of GAS isolates are quite different in different countries. COVID-19 pandemic has already changed our lifestyle. People pay more attention to protective social distance, wearing masks, personal hygiene, and frequent hand washing [24]. Respiratory infection diseases have been reduced dramatically as well as GAS-related respiratory infection diseases. Because of these, isolates collected in our study were much fewer compared with our previous study. Our present study offered insights into antibiotic resistance, virulence genes of GAS under COVID-19 pandemic.

In our present research, male-to-female ratio was 1.65:1. Kim analyzed children suffered scarlet fever in Jeju in Korea between 2002 and 2016. He presented male-to-female ratio was 1.3:1[25]. In Shanghai, during 2011 to 2015, scarlet fever usually affected children aged three to nine [12]. Patients from our present study, aged from 22 days to 11 years old, with median 6.58 years old.

Resistance rate of macrolides in our present study was still high compared with our previous studies from 2016 to 2017, and 2019. Yu found that from 2016 to 2018, 342 GAS strains were highly susceptible to penicillin, levofloxacin, and chloramphenicol, whereas most of strains were resistant to azithromycin, erythromycin, clarithromycin, clindamycin, and tetracycline [9]. Since 1990, the resistance rate of GAS against clindamycin and macrolides has been high [7]. Chinese strains mainly harbored *ermB* gene. In our study, 93.86% stains harbored *ermB* gene. Distribution of *ermB* gene in our GAS strains among scarlet fever, impetigo, psoriasis, allergic purpura, and suppurative tonsillitis was 95.24%, 100%, 80%, 100%, and 100% respectively (Table 3). In our previous study from 2016 to 2017, 97.64% GAS strains harbored *ermB* gene. In the year of 2019, we found 89.67% isolates harbored *ermB* gene.

M protein is immune-dominant GAS protein, locating on surface of bacterial cell wall [26], which adhering to host cell and block phagocytosis, aiding GAS colonization [27]. Macrolide resistance in GAS links to some *emm* types. In our study, *emm12.0* and *emm1.0* were predominant types in macrolide resistance GAS. *Emm12.0* carried *ermB* was the most frequent macrolide resistance isolates, which was consistent with Liang’s study between 2005 and 2008 as well as our study in 2009 [22].

M protein and sAgs play an important role in GAS infection pathogenesis. There is a close relationship between *emm* types and sAgs [28]. In this study, we presented distribution of *emm* types including 13 *emm* types and 11 sAgs. Types *emm12.0* and *emm1.0* exhibited higher polymorphism rate which were similar with our previous study as well as Yu’ study from 2016 to 2018 [9]. They were responsible for about 73.81% of scarlet fever cases in our present study. Tsai collected 320 GAS strains from 339 children in Southern Taiwan. *Emm12* (63.8%) was dominant type, following *emm1* (16.9%), *emm4* (11/0.9%) during 2000 to 2019 [29].

The dominant *emm12.0*, *emm1.0*, *emm12.19*, and *emm12.67* types in this study were similar to those in Southeast Asia, UK and Southern Taiwan [30], but were different from results presented in Portugal and Canada. Ana exhibited markers of invasive GAS were *emm1* and *emm*64, *speA*, and *speJ* independently, However, GAS carried *emm4*, *emm75*, *ssa*, *speL/M* genes were independent markers in pharyngitis [31]. In Canada, since 2010, *emm1* has been the most frequent type. Epidemic scarlet fever has been reported in China, United Kingdom. In China, UK. GAS isolates were *emm1*, *emm12*, *emm3*, and* emm4* respectively carrying *speA*, *speC*, *ssa* [32]. Our research was a little different from previously epidemic reports. *Emm12* strains had been major epidemic isolates.

GAS M protein has been surveillance in Beijing from 2011 to 2018, meanwhile, M 12 stains began to decrease from 2011, and the lowest point was in 2014. Meanwhile, M 1 stains began to raise, and reached to the highest point in 2014, and then exceed M 12 from 2013 to 2014 [33]. However, our present research was different form Yu’ research. During 2019–2021, 2016–2017, we found GAS from scarlet fever and impetigo carried *emm12*, predominantly. In psoriasis, GAS carried *emm1* in 2019 (Table 4), however, between 2020 and 2021, the isolates carried *emm12*, *emm12.29* and *emm89* predominantly [21, 22]. Patricia found *emm*70, *emm*33, *emm*25, *emm*93.3,and *emm*11 were the most frequent emm types among impetigo, pharyngitis, and asymptomatic throat [3].

Liang and Luca found *emm1.0* isolates harbored *speA*, *speC* with similar frequencies, meanwhile, *emm12.0* carried low frequencies *speA*, and high frequencies *speC*. The frequencies of *speA*, *speC* among *emm1.0*, *emm12.0* isolates in present study were consistent with Liang’s results[20], while that in our previous study were in agreement with Luca’s results[34].

In our present study, 11 *sAgs* were detected in GAS isolates. *SmeZ*, *ssa*, *speC* were the most common *sAgs*. *Emm1* carried *speG*, *ssa*, *smeZ*, *speC*, and *speA*. However, content of *speH*, *speI*, and *speM* was less. *Emm12* harbored *speG*, *ssa*, *smeZ* and *speG*, with little *speA*, *speJ* and *speM*. Both *emm1.0* and *emm12.0* had no *speK*, *speL*. Lu found among invasive or not GAS isolates harbored *speB*, and *slo*, meanwile, *smeZ*, *speC*, and *speF* were determined in more than 90% isolates from 2009 to 2016 in 7 cities in China. These isolates carried *emm12.0* (42.9%) and *emm1.0* (30.7%) [35].

Liang found scarlet fever isolates carried *speA* (52.4%), and *speC* (79.3%) from 2005 to 2008 in mainland China [20]. SAg distribution was varied in different geographic areas. In France, Plainvert exhibited GAS strains carried *speA* (59%), *speC* (37%), *ssa* (13%), and *smeZ* (92%) in meningitis from 2003 to 2013. During 2006 to 2009, Friaes presented more than 90% GAS isolates carried *speG* and *smeZ*. In Ireland, Mary exhibited invasive *emm* types were *emm1*, *emm3*, meanwhile, in non-invasive GAS isolates were *emm4*, *emm28*, and *emm3*. *SpeA*, *speG* and *speJ* were related with invasive GAS isolates, whereas *speC*, *speI*, and *ssa* with non-invasive GAS infections [36]. According to our present data, we found scarlet fever isolates harbored *speC* (94.05%), and *smeZ* (98.81%), psoriasis isolates carried *speC* (80%), and *smeZ* (100%). Impetigo isolates carried *speC* (88.89%), *ssa* (88.89%), and *smeZ* (88.89%). In our previous study from 2016 to 2017, we found that the most prevalent scarlet fever isolates carried *smeZ* (96.97%), *speC* (92.59%) and *speG* (91.58%), presented in Table 6. However, in our study of 2019, the most prevalent GAS carried *smeZ* (94.46%), *speC* (91.14%) and *ssa* (74.91%). Scarlet fever isolates prevalently harbored *smeZ* (93.6%), *speC* (90.4%). Psoriasis isolates harbored *smeZ* (100%), *speC* (100%), and impetigo isolates harbored *smeZ* (100%)*, ssa* (89.7%)*, *and* speC* (89.7%) [22]. Catarina collected 303 GAS strains from scarlet fever, tonsilla-pharyngitis patients between 2002 and 2008. Isolates from scarlet fever carried *smeZ*, *ssa*, *speG* and *speC*. Strains from pharyngitis carried *smeZ*, *speG*, *speC*, and *ssa* [37].

**Table 6:** Distribution of superantigens and *emm* types in isolates among different GAS infected cutaneous diseases

*emm* types	Scarlet fever (*n* = 84)							Impetigo (*n* = 17)							Psoriasis (*n* = 10)							Count 114
	*speA*	*speC*	*speH*	*speI*	*SpeJ*	*ssa*	*smeZ*	*speA*	*speC*	*speH*	*speI*	*speJ*	*ssa*	*smeZ*	*speA*	*speC*	*speH*	*speI*	*speJ*	*ssa*	*smeZ*	
*emm1.0*	21	25	4	2	23	25	25	0	1	0	0	1	1	1	1	1	0	0	1	1	1	134
*emm12.0*	2	34	32	31	1	36	36	0	12	11	10	0	12	12	2	2	1	1	2	2	2	241
*emm11.0*	0	0	0	0	0	0	0	0	0	0	0	0	0	0	0	1	1	1	0	0	1	4
*emm12.19*	1	5	0	0	2	5	5	0	0	0	0	0	0	0	0	0	0	0	0	0	0	18
*emm12.21*	1	3	2	2	1	3	3	0	0	0	0	0	0	0	0	0	0	0	0	0	0	15
*emm12.29*	0	1	1	1	0	1	1	0	0	0	0	0	0	0	0	2	2	2	0	2	2	15
*emm12.37*	0	2	2	2	0	2	2	0	1	1	1	0	1	1	0	0	0	0	0	0	0	15
*emm12.65*	0	2	0	1	0	2	2	0	0	0	0	0	0	0	0	0	0	0	0	0	0	7
*emm12.67*	1	2	3	3	0	2	4	0	0	0	0	0	0	0	0	1	1	1	0	1	1	20
*emm12.69*	0	1	0	0	0	1	1	1	1	0	0	1	1	1	0	0	0	0	0	0	0	8
*emm4.0*	0	1	0	0	0	1	1	0	0	0	0	0	0	0	0	0	0	0	0	0	0	3
*emm75.0*	0	2	2	2	0	0	2	0	0	0	0	0	0	0	0	1	1	1	0	0	1	12
*emm89.0*	0	1	0	0	0	0	1	0	1	0	0	0	1	1	0	0	0	0	0	0	2	7
totle	26	79	46	44	27	78	83	1	16	12	11	2	16	16	3	8	6	6	2	6	10	114
Percentage%	30.95	94.05	54.76	52.38	32.14	92.86	98.81	5.55	88.89	66.67	61.11	11.11	88.89	88.98	30	80	60	60	20	60	100	

Our study has several limitations. Firstly, our research was conducted at a single center, which could have made biases to occurrence of GAS infected cutaneous diseases. Under COVID-19 pandemic, outpatients deceased dramatically. Atypical symptoms might have been misdiagnosed. Secondly, our study had small GAS isolates which might not fully represent GAS types under COVID-19 pandemic.

In summary, our study exhibited epidemiology and molecular characteristics of GAS infection cutaneous diseases in a children’ hospital in Beijing under COVID-19 pandemic. We compared our research with researches before COVID-19 pandemic. Collections of GAS infected cutaneous diseases decreased dramatically. M proteins in psoriasis were different in the year of 2019 and 2020 to 2021. There were no significant changes in epidemiology and molecular characteristics of GAS in children with scarlet fever, impetigo before and during COVID-19 pandemic. Long-term surveillance and investigation of *emm* types and superantigens of GAS prevalence are necessary.

## Disclaimer

The study sponsors had no role in study design; collection, analysis, and interpretation of data; writing the report; or the decision to submit the report for publication.

## Data Availability

The datasets generated and/or analyzed during the current study are available from the corresponding author on reasonable request.
